# Effects of different lactic acid bacteria fermentation on active substances and functional characteristics of honeysuckle liquid: analysis of metabolites of honeysuckle liquid based on metabolomics

**DOI:** 10.3389/fmicb.2025.1595351

**Published:** 2025-05-26

**Authors:** Jing Yang, Hanhan Shi, Lili Cai, Sensen Zhang, Yuanzeng Zhao, Xiaojia Su, Haiyan Sun, Yinping Cao, Yongchao Li

**Affiliations:** ^1^School of Life Sciences, Henan Institute of Science and Technology, Henan International Joint Laboratory of Plant Genetic Improvement and Soil Remediation, Xinxiang, China; ^2^College of Grain, Oil and Food Science, Yunnan Vocational College of Mechanical and Electrical Technology, Kunming, China

**Keywords:** characteristics, honeysuckle, lactic acid bacteria, metabolomics, polyphenols

## Abstract

In order to study the effects of lactic acid bacteria on the active substances and functions of honeysuckle, metabolomics was used to analyze the differential metabolites of honeysuckle fermented by lactic acid bacteria. The results showed that *Lactobacillus acidophilus* increased the contents of total phenols, flavonoids and chlorogenic acid by 26.48, 22.59, and 33.57%, respectively, compared with Control Group (CK). The antioxidant capacity and *α*-glucosidase activity of fermented honeysuckle liquid (FLJT) were the strongest, and the scavenging activity and reducing ability of DPPH radical, hydroxyl radical and superoxide anion radical increased by 33.96, 25.60, 58.21 and 39.29%, respectively, compared with unfermented honeysuckle liquid without lactic acid bacteria (LJT). The 991 metabolites with significant differences were screened and enriched into 79 metabolic pathways. The contents of 4-hydroxybenzoic acid, 3, 4-dihydroxyhydrocinnamic acid, chlorogenic acid and 4-hydroxycinnamic acid, which have antioxidant and hypoglycemic activities, and flavonoids in FLJT were increased compared with those in LJT. *Lactobacillus acidophilus* fermentation could promote the synthesis and accumulation of secondary metabolites in honeysuckle liquid. This study provides a theoretical basis for the development of honeysuckle functional products.

## 1 Introduction

Honeysuckle (*Lonicera japonica Thunb*.) has high application value, such as antioxidant, hypoglycemic, antibacterial, antiviral, antipyretic, anti-inflammatory and immunomodulatory properties (Ran et al., 2024a; Yan et al., 2020). Lactic acid bacteria (LAB) are a group of non-spore gram-positive bacteria that ferment sugars to produce organic acids, although pathways might be different (Chen et al., 2019). LAB plays an important role in improving intestinal immune function and reducing the risk of colon cancer, and is often used as a starter in the production of probiotic bacteria dairy products, such as yogurt and cheese (Szutowska, 2020). However, dairy products are usually not suitable for consumers such as vegans, those with lactose intolerance, individuals with high cholesterol, and those allergic to milk proteins. As a result, there has been a sharp increase in the demand for plant-based probiotic foods (Chen et al., 2019). Biotransformation of chemical substances in plant matrix through LAB has become an important trend to provide plant-based probiotic functional drinks as substitutes for individuals who cannot eat dairy products (Ricci et al., 2019; Li et al., 2021). LAB fermentation can improve product quality and nutrition via biotransformation, give products unique flavors, and extend their shelf life (Di Cagno et al., 2011), but also improve antioxidant performance, promote intestinal metabolism, prevent cardiovascular and cerebrovascular diseases, etc. It is also friendly to consumers with high cholesterol and poor digestive system, and has the potential to become functional health food (Di Cagno et al., 2019; Sangija et al., 2022; Yuasa et al., 2021). Therefore, it is of great significance to develop honeysuckle probiotic beverage to meet this kind of functional food. Ran et al. (2024b) optimized the process of honeysuckle beverage with strong antioxidant activity. Different strains of LAB have different metabolic reactions to different food substrates, and have different effects on physical and chemical properties, flavor and biological activities of fermented foods (Wu et al., 2020). Sun et al. (2022) fermented pumpkin juice with 5 kinds strains of LAB. *Lacticaseibacillus paracasei* fermented juice had the highest organic acid content; *Lactobacillus acidophilus* and *Lactobacillus helveticus* fermented juices had the highest sensory scores; and the juices fermented by *Lactiplantibacillus plantarum*, *L. acidophilus*, and *L. helveticus* showed the strongest DPPH and hydroxyl radical scavenging abilities. Quan et al. (2022) fermented orange juice by three LAB, *L. paracasei* fermented orange juice has better aroma and overall acceptability and enhanced antioxidant activity. Liu et al. (2022) found that *L. plantarum* fermented seabuckthorn juice showed the best sensory score and flavor formation, while *Lacticaseibacillus casei* had the highest reducing sugar utilization rate and the highest lactic acid production. Qi et al. (2021) fermented *Lycium barbarum* juice by 6 kinds strains of LAB, the organic acids and bioactive substances by *L. paracasei* and *L. acidophilus* fermented *Lycium barbarum* juice increased significantly.

Metabolomics is a comprehensive analysis method based on metabolites in raw materials, which can be combined with high-throughput detection instruments to obtain high-throughput data and carry out batch correlation analysis, and can analyze low-concentration compounds in raw materials more comprehensively (Zhang et al., 2019). Metabolomics based on multivariate statistics can explore the potential relationship between compounds by analyzing data, obtain the diversity and similarity of compounds in different samples, and then screen out potential markers (Zhuang et al., 2020).

In order to further study the fermentation mechanism and understand the differences of metabolites of honeysuckle before and after fermentation, this study analyzed the effects of different strains of LAB on the color characteristics, bioactive substances, antioxidant activity and hypoglycemic activity of honeysuckle liquid, screened out the best fermentation strains, and analyzed the changes of metabolic components of honeysuckle liquid before and after fermentation by metabolomics technology.

## 2 Materials and methods

### 2.1 Materials and reagents

Honeysuckle was obtained from Yanjin Honeysuckle Experimental Base of Henan Institute of Science and Technology.

Gallic acid (CAS 149-91-7), Chengdu Manster Co., Ltd.; Rutin (CAS 153-18-4), Folin & Ciocalteu’s phenol reagent (Product code F8060), Beijing Solaibao Co., Ltd.; Chlorogenic acid (CAS 327-97-9), Sigma-Aldrich Company, USA; Formic acid (CAS 64-18-6), methanol (chromatographic pure) (CAS 67-56-1), Xilong Science Co., Ltd.; Other reagents are analytically pure.

### 2.2 Strains and culture

*Lacticaseibacillus rhamnosus* zrx01 (*L. rhamnosus* GG zrx01, LGG), *Lactobacillus acidophilus* zrx02 (*L. acidophilus* zrx02, LA), *Lactiplantibacillus plantarum* zrx03 (*L. plantarum* zrx03, LP-1), *Pediococcus pentosus* Mi515 (*P. pentosaceus* Mi 515, PP), *Lactobacillus fermentans* 3.2 (*L. fermentum* 3.2, LF), *Lactiplantibacillus plantarum* CN2018 (*L. plantarum* CN2018, LP-2), *Lactiplantibacillus plantarum* LP45 (*L. plantarum* LP45, LP-3), *Bifidobacterium* zrx04 (*Bifidobacterium* zrx04, B), *Streptococcus salivarius* subsp. *thermophilus* zrx05 (*S. thermophilus* zrx05, ST), *Lactobacillus delbruechii* subsp. *bulgaria* zrx06 (*L. delbrueckii* subsp. *bulgaricus* zrx06, LB) were stored in the laboratory.

First, 100 μL of each bacterial solution was inoculated into 10 mL of MRS medium and incubated at 37°C for 12 h. Subsequently, 1 mL of the culture was transferred to 100 mL of MRS medium and cultured again at 37°C for 12 h. The bacteria were harvested by centrifugation at 6000 × g for 10 min at 4°C, washed twice with an equal volume of sterile 0.9% NaCl solution, and resuspended in 1 mL of sterile water for further use.

### 2.3 Preparation and fermentation of honeysuckle liquid

Honeysuckle was added to distilled water at a ratio of 3 g/100 g, and sucrose was incorporated as the carbon source at 7 g/100 g. The mixture was then pasteurized at 60°C for 30 min prior to fermentation. Microbiological analysis confirmed the absence of LAB in both the raw and pasteurized honeysuckle liquid. Once cooled to room temperature, 3 mL of each strain’s bacterial suspension was added per 100 mL of pasteurized honeysuckle liquid. Fermentation occurred anaerobically at 37°C for 24 h, with three replicates conducted for each treatment. Before fermentation, the inoculation concentrations (IC) of the ten LAB strains were standardized to 7.46 ± 0.28 log CFU/mL. Unfermented honeysuckle liquid without LAB (LJT) served as the control group.

### 2.4 Determination of viable bacteria number and physical and chemical properties

According to the method of Wang et al. (2022), the viable bacteria number and physical and chemical properties of honeysuckle liquid were determined, three replicates. The number of LAB viable bacteria was determined on MRS agar plate. The samples were continuously diluted to 10^−5^–10^−8^ with sterile normal saline. The 100 μL diluted samples were uniformly spread onto MRS agar plate and cultured in 37°C incubator for 36–48 h. Then, the number of cells containing 30–300 colonies on the plate was measured and recorded as log CFU/mL. The pH value of the sample was measured directly by using the calibrated pH meter. Hunter colorimeter was used to determine the color characteristics of honeysuckle liquid (L*, a* and b*). L* denotes brightness, and the higher the L value, the more white the color is. a* indicates red-green color, positive value indicates that the color is biased toward red, and negative value indicates that the color is biased toward green; b* denotes yellowish blue, a positive value denotes a color bias toward yellow, and a negative value denotes a color bias toward blue. ΔE represents the total color difference of FLJT compared with LJT. The larger the ΔE value, the more obvious the color difference is. ΔE is calculated according to following formula:


$$\Delta E=\sqrt{{\left(L_{0}*-L*\right)}^{2}+{\left(a_{0}*-a*\right)}^{2}+{\left(b_{0}*-b*\right)}^{2}}$$

L_0_*, a_0_*, and b_0_*, respectively, represent the values of L, a, and b in the control group, and L*, a*, and b*, respectively, represent the values of L, a, and b in the fermentation group.

### 2.5 Determination of bioactive substances

#### 2.5.1 Total phenol content: Folin–Ciocalteu method

A volume of 1 mL of the 10-fold diluted sample was mixed with 5 mL of 10% Folin - Ciocalteu reagent in a 50 mL Erlenmeyer flask. The mixture was allowed to react for 5 min, followed by the addition of 4 mL of Na₂CO₃ solution (75 g/L). After further incubation for 40 min, the absorbance was measured at 765 nm. The results were presented as gallic acid equivalent (mg GAE/100 mL) (Zhou et al., 2020).

#### 2.5.2 Total flavonoids content: AlCl_3_ colorimetry

A volume of 4 mL of the 10 - fold diluted sample was mixed with 0.5 mL of NaNO₂ solution (50 g/L) in a 10 mL tube and allowed to stand for 5 min. Then, 1 mL of AlCl₃ solution (100 g/L) was added, and the mixture was reacted for another 5 min. Subsequently, 2 mL of NaOH solution (2 mol/L) was added, and the reaction was carried out for 10 min. Finally, the absorbance was measured at 510 nm. The results were expressed as rutin equivalent (mg RE/100 mL) (Kwaw et al., 2018).

#### 2.5.3 Chlorogenic acid content: HPLC

A volume of 1 mL sample that had been diluted 10 times was centrifuged at 4°C for 15 min at 7000 × g, the supernatant was filtered with a 0.22 um filter membrane and injected into a high performance liquid chromatography system (Waters Inc., USA) on a TC-C18 column 250 mm × 4.6 mm, 5 um (Agilent, USA); Temperature 30°C; The detection wavelength is 320 nm; Injection volume 20 μL; Volume flow rate is 0.6 mL/min; The mobile phase is 0.1% formic acid aqueous solution (A) and methanol solution (B); The gradient elution condition is 0–10 min, 5–30% B; 10–25 min, 30–70% B; 25–35 min, 50–70% B; 35–40 min, 70–5% B. The results were expressed as chlorogenic acid equivalent (mg CGA/100 mL) (Oteef, 2022).

### 2.6 Determination of antioxidant activity

#### 2.6.1 DPPH radical scavenging activity

The method of Qi et al. (2021) was referred with some modifications. A volume of 2 mL sample diluted 5 times or distilled water (control) was added to 4 mL DPPH solution (0.2 mmol/L) for 30 min, and the absorbance was measured at 517 nm. DPPH radical scavenging activity is calculated according to following formula:


$$\;\text{DPPH radical scavenging activity}\;(\%)=\frac{A_{\text{control}}-A_{\text{sample}}}{A_{\text{control}}}\times 100$$

A control refers to replacing the absorbance of the sample with distilled water; A sample is the absorbance of the sample.

#### 2.6.2 Hydroxyl radical scavenging activity

The method of Zhang et al. (2021) was referred with some modifications. A volume of 1 mL of 2-fold diluted sample or distilled water (control) was added to 1 mL of 1, 10-phenanthroline solution (0.75 mmol/L) and 2 mL of sodium phosphate buffer (PBS, pH 7.4). After mixing, 1 mL of FeSO_4_ solution (0.75 mmol/L) and 1 mL of H_2_O_2_ solution (0.12%, v/v) were added. The reaction was carried out at 37°C for 30 min, and the absorbance was measured at 536 nm. Hydroxyl radical scavenging activity is calculated according to following formula:


$$\cdot \mathrm{OH}\;\text{radical scavenging activity}\;(\%)=\frac{A_{\text{sample}}-A_{\text{blank}}}{A_{\text{control}}-A_{\text{blank}}}\times 100$$

A control refers to replacing the absorbance of the sample with distilled water; A sample refers to the absorbance of the sample; A blank refers to the absorbance of a sample with distilled water instead of H_2_O_2_.

#### 2.6.3 Superoxide anion radical scavenging activity

Pyrogallol autoxidation method was used to determine superoxide anion radical scavenging activity (Mei et al., 2020). The 1 mL sample solution or distilled water (control) was added to 2.4 mL distilled water, add 4.5 mL Tris–HCl buffer (0.1 mmol/L, pH = 8.2), mix and add 0.1 mL pyrogallol solution (30 mmol/L), and measure the absorbance at 325 nm. Superoxide anion radical scavenging activity is calculated according to following formula:


$$O_{2}^{-}\;\text{radical scavenging activity}\;(\%)=\frac{A_{\text{control}}-A_{\text{sample}}}{A_{\text{control}}}\times 100$$

A control refers to replacing the absorbance of the sample with distilled water; A sample is the absorbance of the sample.

#### 2.6.4 Determination of reducing ability

The method of Lu et al. (2018) was referred with some modifications. A volume of 0.5 mL of sample was added to 2.5 mL of phosphate buffer (0.2 mol/L, pH 6.6) and 2.5 mL of 1% potassium ferricyanide solution for 20 min at 50°C, then 2.5 mL of 10% trichloroacetic acid solution was added, centrifuged for 10 min (3,500 × g), a volume of 2.5 mL of supernatant was mixed with 2.5 mL of distilled water and 0.5 mL of 0.1% ferric chloride solution, and then the absorbance was measured at 700 nm. Higher absorbance means stronger reducing ability.

### 2.7 *α*-Glucosidase inhibitory capacity

The method of Zhang et al. (2021) was refer with some modifications. A volume of 100 μL sample that had been diluted 10 times or distilled water (control) was added to 100 μL alpha-glucosidase solution (0.5 U/mL) and reacted at 37°C for 10 min, then 100 μL pNP-G solution (5 mmol/L) was added for 20 min, and the absorbance was recorded with a microplate reader at 405 nm. The α -glucosidase inhibitory capacity is calculated according to following formula:


$$\alpha -\text{glucosidase inhibitory capacity}\;(\%)=\frac{A_{\text{control}}-A_{\text{sample}}}{A_{\text{control}}}\times 100$$

A control refers to replacing the absorbance of the sample with distilled water, and A sample refers to the absorbance of the sample.

### 2.8 Sample information

The test samples were shown in Table 1. The unfermented honeysuckle liquid without LAB (LJT) and *L. acidophilus* zrx02 fermented honeysuckle liquid (FLJT) were selected for metabolic research. At the same time, quality control (QC) was prepared to investigate the stability of equilibrium chromatography-mass spectrometry system and detection process, so as to ensure the reliability of the results.

**Table 1:** Sample information.

Group	Sample group name	Tag name
1	LJT	LJT1, LJT2, LJT3
2	FLJT	FLJT1, FLJT2, FLJT3

### 2.9 Sample extraction

Samples were taken from −80°C refrigerator, thawed at 4°C, swirled for 10 s and mixed evenly. A volume of 100 *μ*L samples were added into a corresponding numbered 1.5 mL centrifuge tube, 100 μL 70% methanol with internal standard extract was added, swirled for 15 min, then centrifuged for 3 min (9,600 × g, 4°C), and the supernatant was obtained. The supernatant was filtered with a microporous membrane (0.22 μm) and stored in an injection bottle for LC–MS/MS.

### 2.10 Conditions of chromatography and mass spectrometry

#### 2.10.1 Chromatographic conditions

UPLC system Waters ACQUITY UPLC HSS T3 column (1.8 μ m, 2.1 mm × 100 mm) for separation; Column temperature 40°C; Flow rate 0.4 mL/min; Injection quantity 4 μL; Mobile phase A: ultrapure water (0.1% formic acid); Mobile phase B: acetonitrile (0.1% formic acid); Gradient conditions of mobile phase: 0–5 min, 5–65% B; 5–6 min, 65–99% B; The mobile phase B was maintained at 99% in 6–7.5 min; 7.5–7.6 min, 99–5% B; 7.6–10 min, the mobile phase B maintained at 5%; Samples were placed in a 4°C automatic sampler throughout the analysis (Zhang et al., 2019; Zhuang et al., 2020).

#### 2.10.2 Mass spectrometry conditions

Electrospray ionization (ESI) positive ion and negative ion mode were used to detect each sample. Samples were separated by UPLC system and analyzed by TripleTOF 6,600 mass spectrometer. ESI source and mass spectrometry conditions: first-order mass scanning range (m/z): 50–1,250; Positive mode ionization voltage (kV): +5.0, negative mode ionization voltage (kV): −4.0; Ion source temperature 550°C; Spray pressure (psi): 50; Auxiliary heating pressure (psi): 60; Air curtain pressure (psi), 35; Positive mode de-clustering voltage (V): +60, negative mode de-clustering voltage (V): −60; Collision energy (eV): 15.

The original data file acquisited by LC–MS was converted into mzXML format by ProteoWizard software. Peak extraction, peak alignment and retention time correction were, respectively, performed by XCMS program. The “SVR” method was used to correct the peak area. The peaks with detetion rate lower than 50% in each group of samples were discarded. After that, metabolic identification information was obtained by searching the laboratory’s self-built database, integrated public database, AI database and metDNA.

### 2.11 Data analysis

All experiments were subjected to three replicate experiments, and each experiment was measured three times. The results were expressed by mean ± standard deviation. SPSS 20.0 was used for one-way analysis of variance (ANOVA). *p* < 0.05 was statistically significant. The original data of mass spectrometry is converted into mzXmL format by ProteoWizard. First, baseline filtering, peak extraction, peak alignment and retention time correction are carried out by XCMS software. Then, the samples with peak missing rate > 50% are filtered, and the blank values are filled with KNN, and the peak area is corrected by SVR method. Metabolite identification, data preprocessing and quality evaluation were carried out on the corrected and screened peaks, and then data analysis was carried out. Principal component analysis (PCA) was used to observe the overall metabolic status and stability in the analysis process, then partial least squares discriminant analysis (PLS-DA) was used to identify the metabolic differences between groups, orthogonal partial least squares discriminant analysis (OPLS-DA) was constructed to further distinguish and screen the metabolites which contributed greatly to the metabolic differences between groups, and KEGG database was used to analyze the metabolomics pathway.

## 3 Results

### 3.1 Viable number, pH, and color characteristics of LAB in fermented honeysuckle liquid

**Figure 1:**
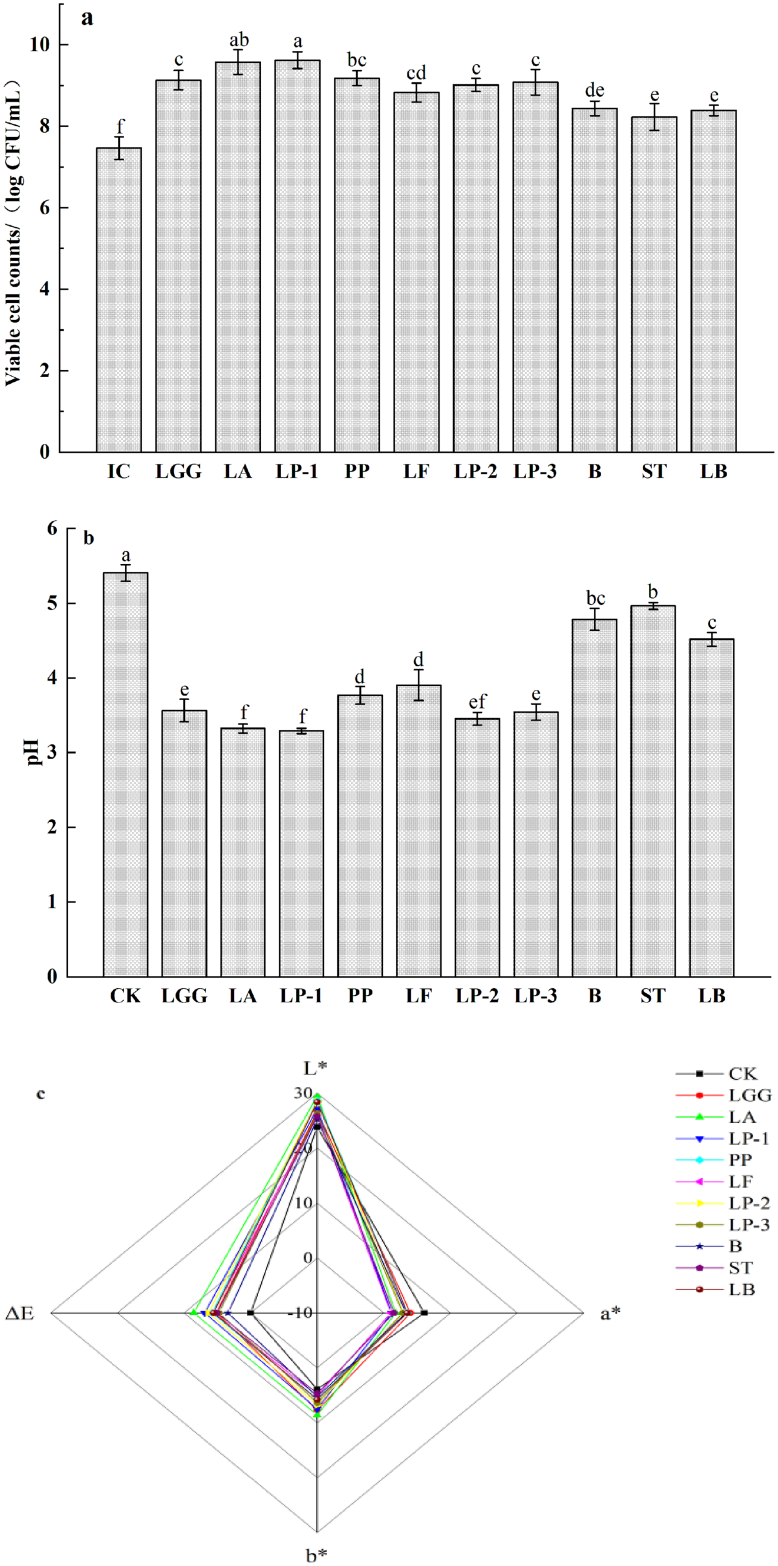
Effect of different LAB fermentation on the count of viable bacteria **(a)**, pH **(b)**, and chroma **(c)** of honeysuckle liquid. *Lacticaseibacillus rhamnosus* zrx01 (LGG), *Lactobacillus acidophilus* zrx02 (LA), *Lactiplantibacillus plantarum* zrx03 (LP-1), *Pediococcus pentosus* Mi515 (PP), *Lactobacillus fermentans* 3.2 (LF), *Lactiplantibacillus plantarum* CN2018 (LP-2), *Lactiplantibacillus plantarum* LP45 (LP-3), *Bifidobacterium* zrx04 (B), *Streptococcus salivarius* subsp. *thermophilus* zrx05 (ST), *Lactobacillus delbruechii* subsp. *bulgaria* zrx06 (LB). Different lowercase letters indicated that there was a significant difference. The data presented are the averages of 3 replicates.

As shown in Figure 1a, the inoculation concentrations (IC) of the 10 strains of LAB were adjusted to 7.46 ± 0.28 log CFU/mL, the number of viable bacteria of 10 strains of LAB increased significantly after fermentation (*p* < 0.05). Among them, the number of viable bacteria in honeysuckle broth fermented by LP-1 and LA was higher, which was 9.62 ± 0.21 and 9.57 ± 0.31 log CFU/mL, respectively. The number of viable bacteria in honeysuckle broth fermented by PP, LGG, LP-3 and LP-2 also reached more than 9 log CFU/mL. In addition, the number of viable bacteria in honeysuckle broth fermented by B, LB and ST reached more than 8 log CFU/mL, which were 8.44 ± 0.18, 8.39 ± 0.13 and 8.23 ± 0.33 log CFU/mL. The results indicated that honeysuckle liquid with added sucrose was a good substrate for the growth and proliferation of LAB. As shown in Figure 1b, the pH of fermented honeysuckle liquid decreased significantly from 5.41 to 3.29–4.96. The pH of honeysuckle liquid fermented by LP-1 and LA was the lowest. It can be seen that LP-1 and LA had strong ability to produce acid by using carbon source in honeysuckle liquid. Compared with the control, the L* and B* values of the fermented honeysuckle liquid increased and the A* value decreased, the brightness and yellowness of the fermented honeysuckle liquid increased and the redness decreased (Figure 1c).

### 3.2 Content of bioactive substances in fermented honeysuckle liquid

**Figure 2:**
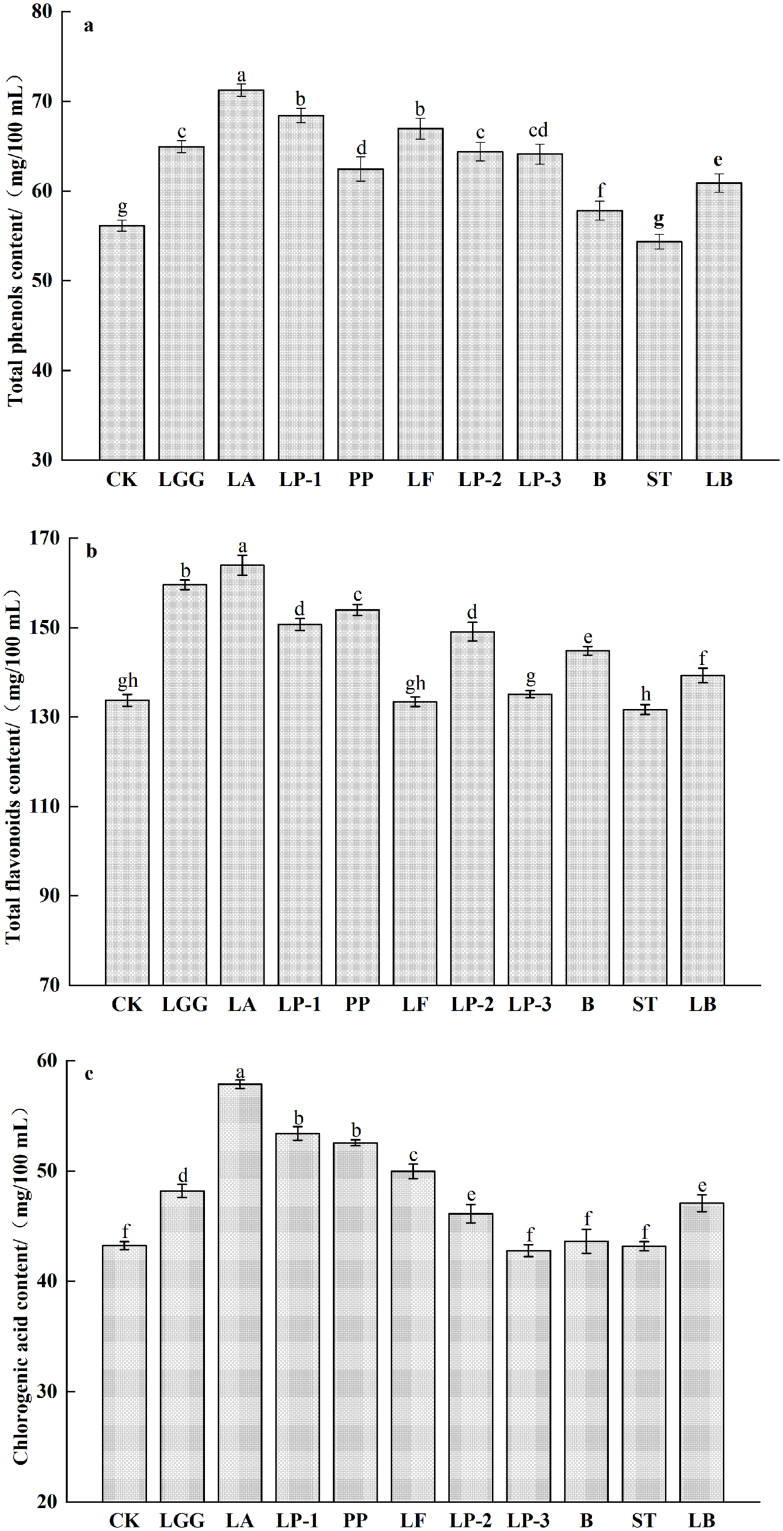
Effect of different LAB fermentation on the total phenols content **(a)**, total flavonoids content **(b)**, and chlorogenic acid content of honeysuckle liquid **(c)**. *Lacticaseibacillus rhamnosus* zrx01 (LGG), *Lactobacillus acidophilus* zrx02 (LA), *Lactiplantibacillus plantarum* zrx03 (LP-1), *Pediococcus pentosus* Mi515 (PP), *Lactobacillus fermentans* 3.2 (LF), *Lactiplantibacillus plantarum* CN2018 (LP-2), *Lactiplantibacillus plantarum* LP45 (LP-3), *Bifidobacterium* zrx04 (B), *Streptococcus salivarius* subsp. *thermophilus* zrx05 (ST), *Lactobacillus delbruechii* subsp. *bulgaria* zrx06 (LB). Different lowercase letters indicated that there was a significant difference. The data presented are the averages of 3 replicates.

The total phenols content of honeysuckle broth fermented by other 9 kinds strains of LAB other than ST significantly increased compared to the control (*p <* 0.05) (Figure 2a). Among them, the total phenols content of honeysuckle liquid fermented by LA was the highest and increased by 26.48% compared with that before fermentation. The total flavonoid content in honeysuckle broth fermented by LF and LP-3 showed no significant change (*p* > 0.05). In contrast, fermentation with ST led to a 1.56% decrease (*p* < 0.05), whereas the remaining 7 kinds strains of LAB significantly increased the total flavonoid content (*p* < 0.05) (Figure 2b). Among them, the content of total flavonoids in honeysuckle liquid fermented by LA was the highest and increased by 22.59% compared with that before fermentation. After LAB fermentation, there was no significant change in chlorogenic acid content of honeysuckle liquid fermented by LP-3, ST and B (*p* > 0.05), while the other 7 kinds strains of LAB increased the chlorogenic acid content of honeysuckle liquid (*p* < 0.05), and the chlorogenic acid content of honeysuckle liquid fermented by LA was the highest, reaching 58.89 mg/100 mL, which increased by 33.57% compared with the control group (Figure 2c).

### 3.3 Antioxidant activity of fermented honeysuckle liquid

**Figure 3:**
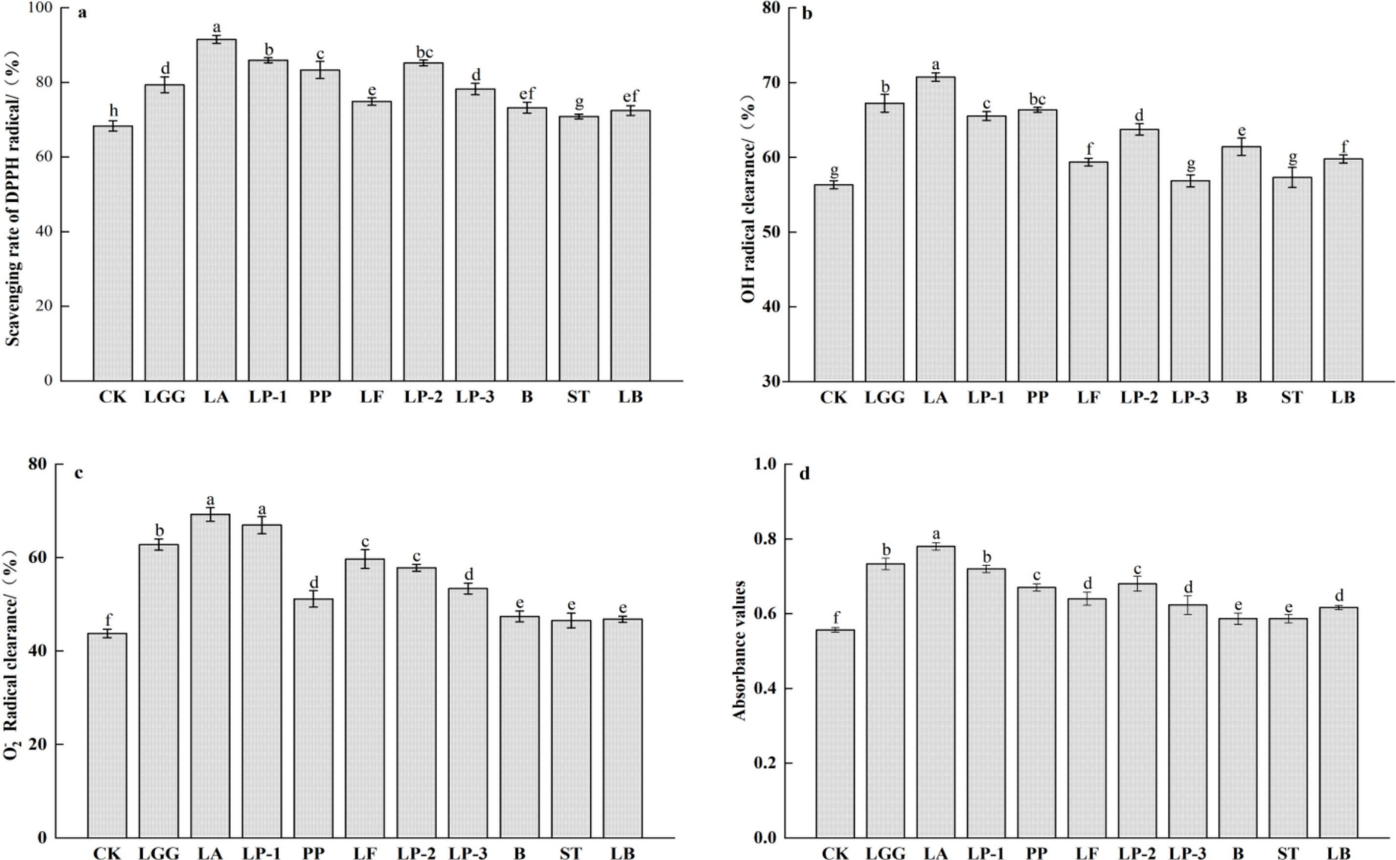
Effect of different LAB fermentation on scavenging rate of DPPH radical **(a)**, OH radical clearance **(b)**, O_2_^−^ radical clearance **(c)**, and reducing ability **(d)** of honeysuckle liquid. *Lacticaseibacillus rhamnosus* zrx01 (LGG), *Lactobacillus acidophilus* zrx02 (LA), *Lactiplantibacillus plantarum* zrx03 (LP-1), *Pediococcus pentosus* Mi515 (PP), *Lactobacillus fermentans* 3.2 (LF), *Lactiplantibacillus plantarum* CN2018 (LP-2), *Lactiplantibacillus plantarum* LP45 (LP-3), *Bifidobacterium* zrx04 (B), *Streptococcus salivarius* subsp. *thermophilus* zrx05 (ST), *Lactobacillus delbruechii* subsp. *bulgaria* zrx06 (LB). Different lowercase letters indicated that there was a significant difference. The data presented are the averages of 3 replicates.

The antioxidant activity of honeysuckle liquid was evaluated using methods for determining DPPH radical, hydroxyl radical, and superoxide anion radical scavenging abilities, as well as reducing ability. Compared with the control group, the scavenging and reducing abilities of DPPH radical, hydroxyl radical, and superoxide anion radical in the honeysuckle liquid fermented by lactic acid bacteria (LAB) were enhanced to different extents (Figure 3). Especially, the scavenging ability of DPPH radical, hydroxyl radical and superoxide anion radical of honeysuckle liquid fermented by LA was the highest, which were 91.52, 70.74 and 69.25% respectively, and the reducing ability of honeysuckle liquid fermented by LA was also the strongest, which increased by 33.96, 25.60, 58.21 and 39.29%, respectively, compared with the control group.

### 3.4 Inhibitory ability of *α*-glucosidase of fermented honeysuckle liquid

**Figure 4:**
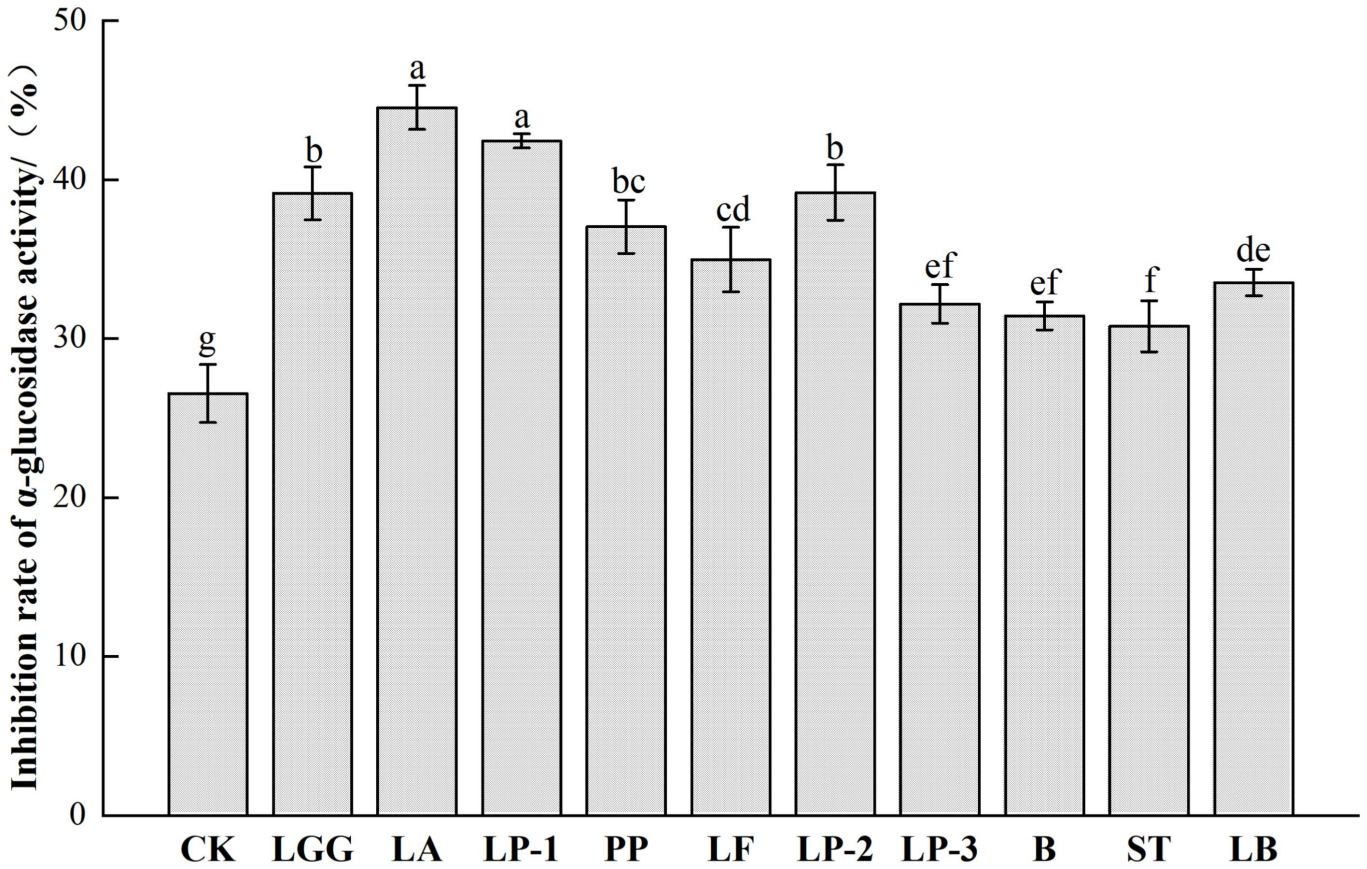
Effect of different LAB fermentation on *α*-glucosidase inhibition of honeysuckle liquid. *Lacticaseibacillus rhamnosus* zrx01 (LGG), *Lactobacillus acidophilus* zrx02 (LA), *Lactiplantibacillus plantarum* zrx03 (LP-1), *Pediococcus pentosus* Mi515 (PP), *Lactobacillus fermentans* 3.2 (LF), *Lactiplantibacillus plantarum* CN2018 (LP-2), *Lactiplantibacillus plantarum* LP45 (LP-3), *Bifidobacterium* zrx04 (B), *Streptococcus salivarius* subsp. *thermophilus* zrx05 (ST), *Lactobacillus delbruechii* subsp. *bulgaria* zrx06 (LB). Different lowercase letters indicated that there was a significant difference. The data presented are the averages of 3 replicates.

The inhibition ability of LAB fermented honeysuckle broth to α-glucosidase was significantly improved, from 26.55% in the control group to 30.78–44.55% (Figure 4). LA-fermented honeysuckle liquid had the strongest inhibition effect on α-glucosidase activity, the inhibition rate was 44.55%, which increased by 67.80% compared with CK group.

### 3.5 Correlation analysis between active substances and functional characteristics of honeysuckle liquid after fermentation

The results were shown in Table 2. Total phenol content was positively correlated with DPPH radical scavenging activity (*r* = 0.834, *p* < 0.01), superoxide anion radical scavenging activity (*r* = 0.935, *p* < 0.01), reducing ability (*r* = 0.870, *p* < 0.01) and α-glucosidase inhibitory activity (*r* = 0.874, *p* < 0.01), and with ·OH radical scavenging activity (*r* = 0.688, *p* < 0.05). Flavonoids content was positively correlated with DPPH radical scavenging activity (*r* = 0.812, *p* < 0.01), hydroxyl radical scavenging activity (*r* = 0.977, *p* < 0.01), reducing ability (*r* = 0.877, *p* < 0.01) and α-glucosidase inhibitory activity (*r* = 0.832, *p* < 0.01), and with superoxide anion radical scavenging activity (*r* = 0.683, *p* < 0.05). Chlorogenic acid content was positively correlated with DPPH radical, hydroxyl radical and superoxide anion radical scavenging activity, reducing ability and α-glucosidase inhibitory ability (*p* < 0.01), and the correlation coefficients were (*r* = 0.777, *r* = 0.831, *r* = 0.761, *r* = 0.837, *r* = 0.843), respectively.

**Table 2:** Pearson’s correlation coefficients of functional properties and bioactive components of FLJT extract.

	Project							
	TPC	TAC	AGC	DPPH	OH	O_2_^−^	RP	α-G
TPC	1.000							
TAC	0.554	1.000						
AGC	0.779^**^	0.700^*^	1.000					
DPPH	0.791^**^	0.788^**^	0.760^**^	1.000				
·OH	0.620	0.978^**^	0.818^**^	0.850^**^	1.000			
O_2_^−^	0.920^**^	0.634^*^	0.738^*^	0.788^**^	0.698^*^	1.000		
RP	0.837^**^	0.865^**^	0.830^**^	0.877^**^	0.900^**^	0.904^**^	1.000	
α-G	0.842^**^	0.826^**^	0.860^**^	0.914^**^	0.885^**^	0.898^**^	0.970^**^	1.000

*p < 0.05; **p < 0.01. TPC, Total phenol content; TAC, Total flavonoid content; AGC, Chlorogenic acid content; DPPH, DPPH free radical scavenging ability; OH, Hydroxyl radical scavenging ability; O_2_^−^, Superoxide anion radical scavenging ability; RP, Reduction ability; α-G, α-glucosidase activity inhibition ability.

### 3.6 Quality control analysis of samples

**Figure 5:**
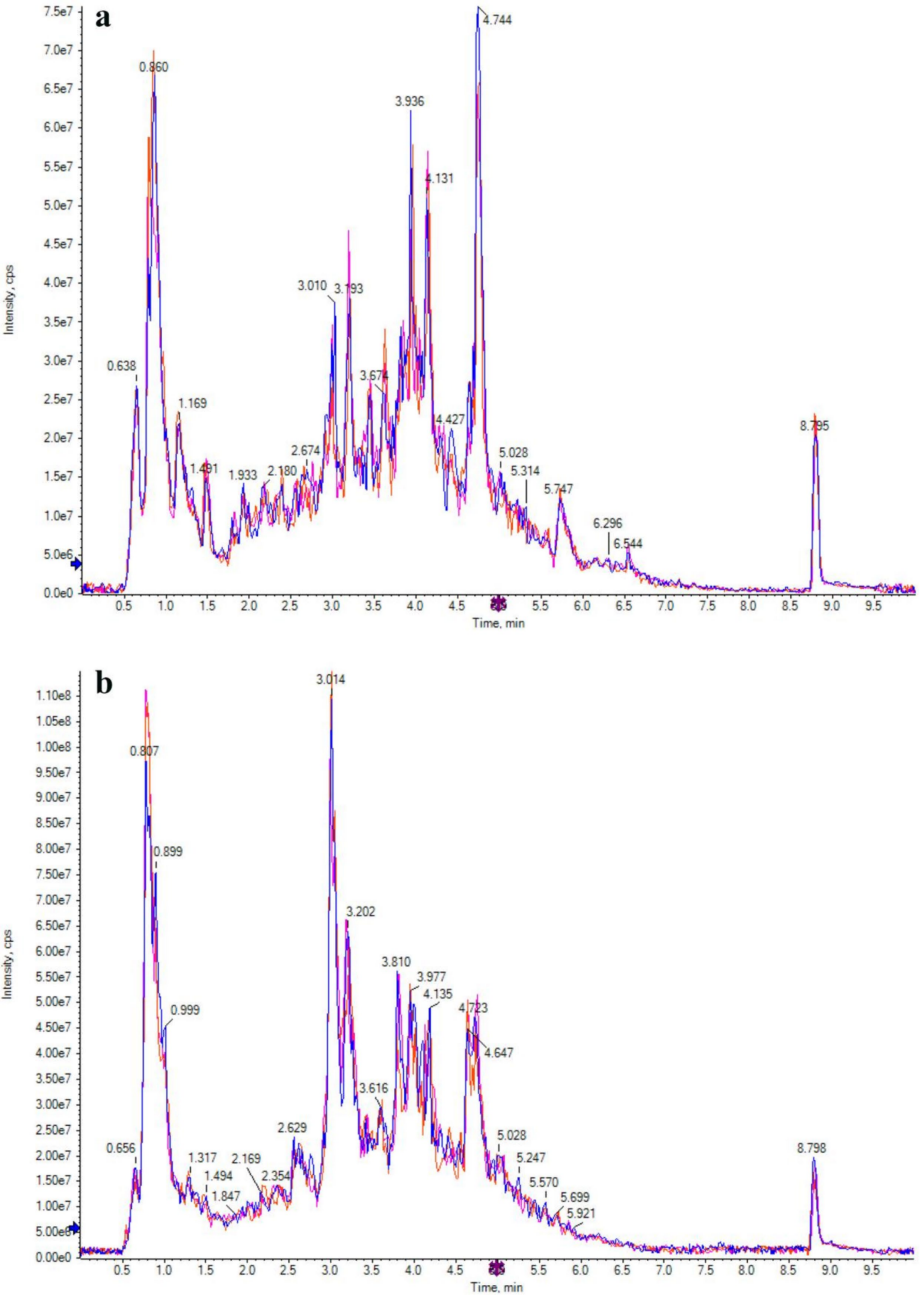
Ion flow chromatogram of positive **(a)** and negative **(b)** ion modes of QC sample.

**Figure 6:**
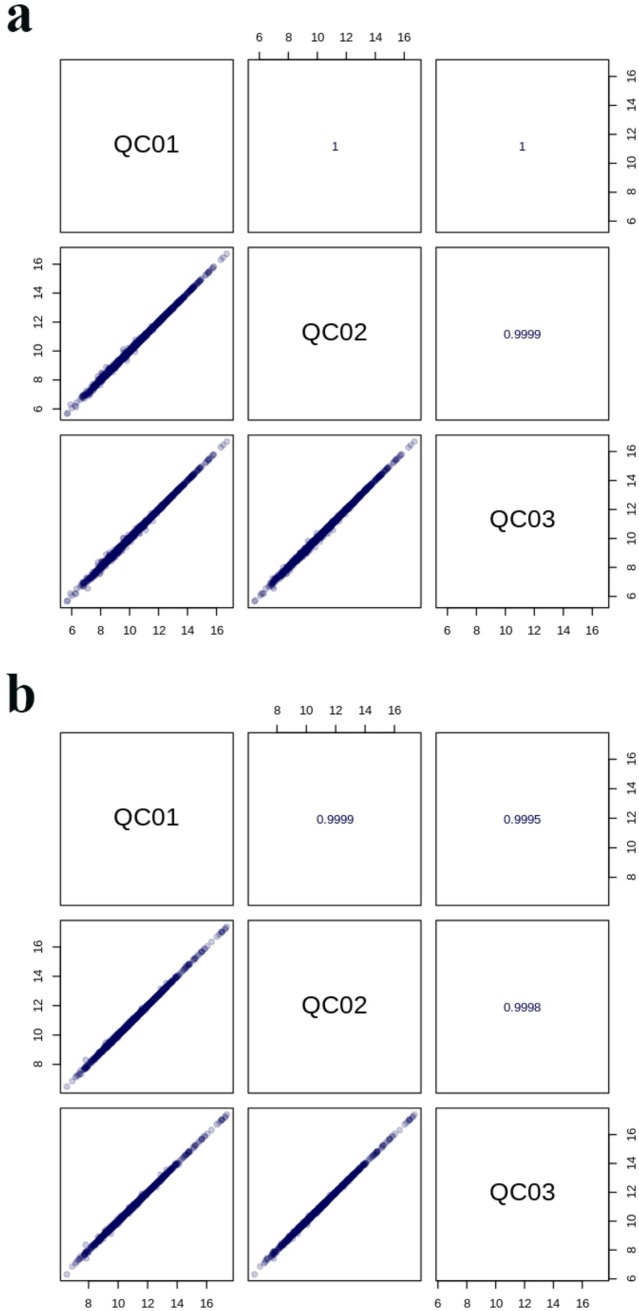
Correlation diagram of positive **(a)** and negative **(b)** ion modes of QC samples.

The TIC patterns of QC samples in positive and negative ion modes were compared by spectral superposition, as shown in Figure 5. As depicted in the figure, QC samples exhibited excellent peak shapes with uniform distribution, and the TIC curves from metabolite detection showed a high degree of overlap. This indicated consistent retention times and peak intensities, suggesting robust signal stability of the mass spectrometry. Consequently, the data demonstrated high reliability and repeatability when detecting the same sample at various time points. Pearson correlation analysis was carried out on QC samples (Figure 6). The higher the correlation of QC samples (R is closer to 1), the better the stability of the whole detection process and the higher the data quality. From the correlation analysis results of positive ion and negative ion mode QC samples in Figure 6, it can be seen that QC samples had high correlation and high data quality.

### 3.7 Overall analysis of metabolites of honeysuckle liquid before and after fermentation

**Figure 7:**
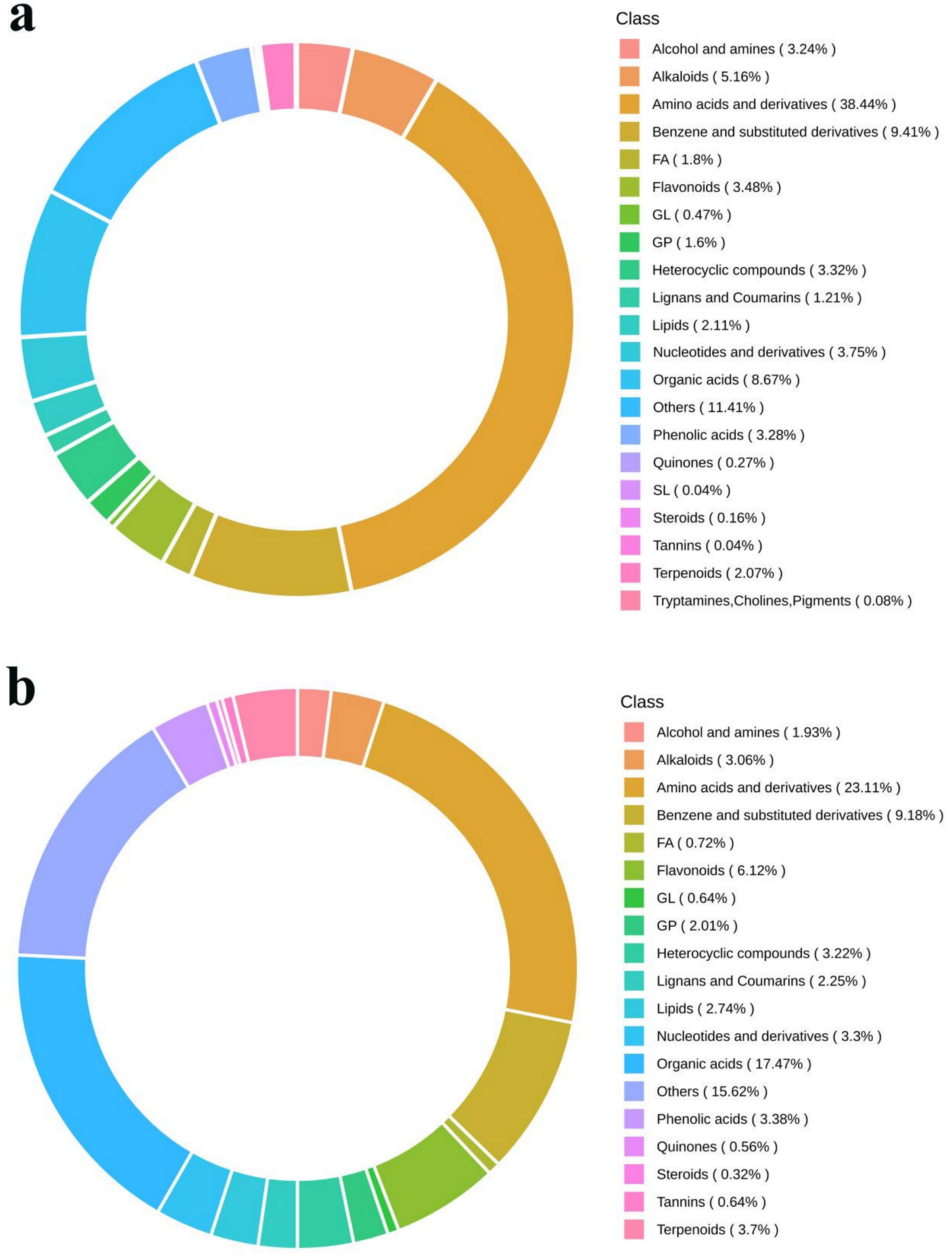
Circular diagram of metabolite category composition of LJT **(a)** and FLJT **(b)**.

The composition of metabolites in samples was specific, and different types of samples had different types and proportions of metabolites. If the treatment methods or biological processes were different, the composition of metabolites would been changed accordingly. Therefore, by analyzing the compositional ratios of metabolites, a comprehensive comprehension of the distribution patterns of the major metabolites in the sample can be obtained. For the metabolism study, the honeysuckle liquid was divided into two groups before and after fermentation. In total, 3,452 metabolites were detected, among which 2,353 metabolites were detected in the positive ion mode and 1,099 metabolites were detected in the negative ion mode (Table 3). As shown in Figure 7, metabolites can be divided into 21 different categories, including 1,172 amino acids and their derivatives, 410 organic acids, 331 benzene and its derivatives, 157 alkaloids, 135 flavonoids, 119 nucleotides and their derivatives, 112 phenolic acids, 110 heterocyclic compounds, 99 alcohols and amines, 63 glycerophospholipids, 20 glycerides, 13 quinones, 49 lignans and coumarins, 1 sphingolipid, 8 tannins, 2 tryptamine, 82 terpenoids, 8 steroids, 51 fatty acyl, 78 lipids, and 432 other compounds. Amino acids and their derivatives (33.95%), organic acids (11.88%), benzene and its derivatives (9.59%), alkaloids (4.59%), flavonoids (3.91%), nucleotides and their derivatives (3.45%) and phenolic acids (3.24%) were the seven dominant metabolites.

**Table 3:** Statistical table of the number of identified metabolites.

Ion mode	Grand total	Positive ion mode	Negative ion mode
Number of metabolites	3,452	2,353	1,099

### 3.8 PCA of metabolites of honeysuckle liquid before and after fermentation

**Figure 8:**
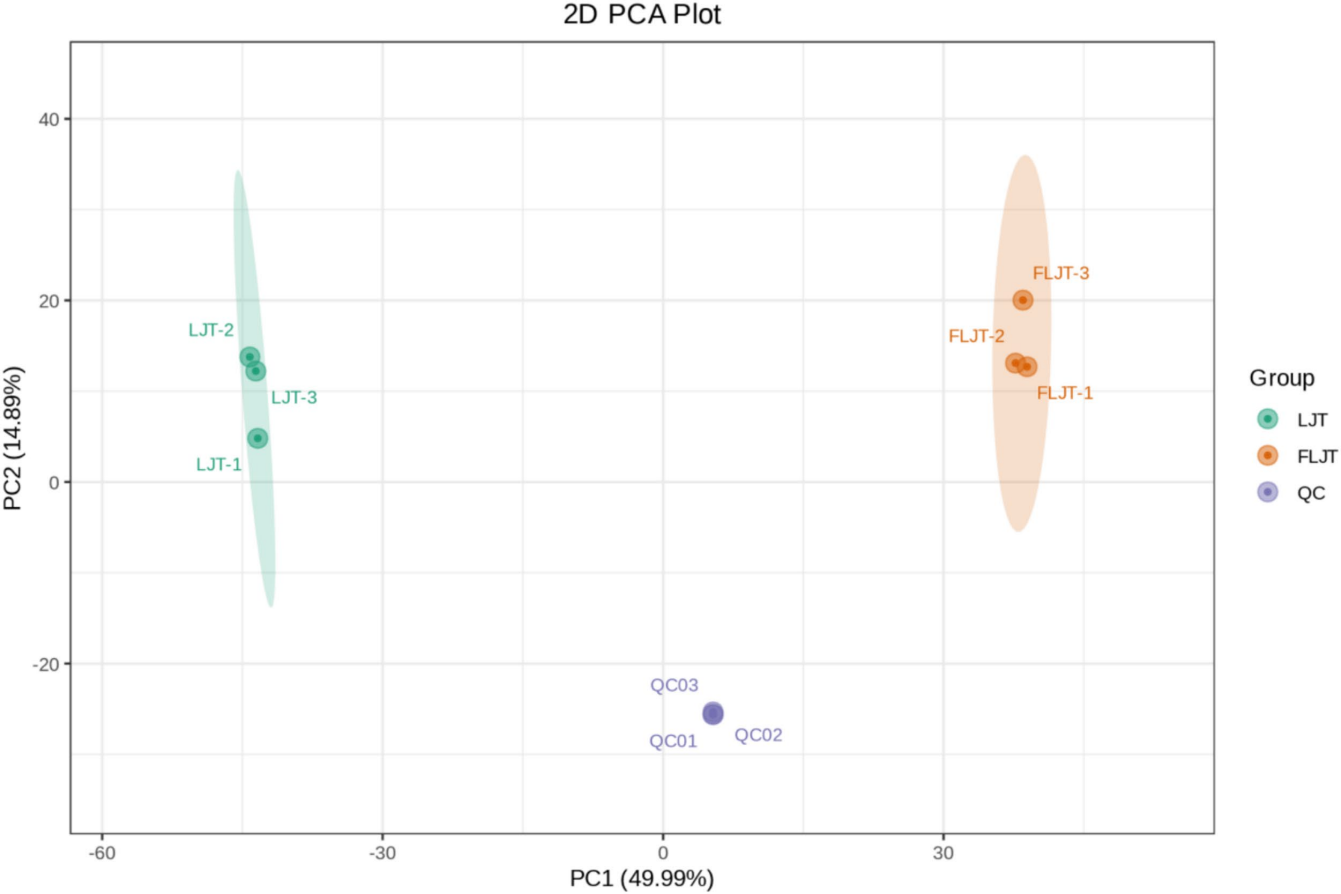
PCA score plot of metabolites of LJT and FLJT.

Principal component analysis (PCA), an unsupervised dimensionality reduction technique, enables an overall observation of the distribution of each sample group. The convergence and dispersion degrees of PCA can reflect the separation tendency and the degree of metabolic differences among different samples, which is conducive to discerning the inter-group differences. That was, on the score plot, the more concentrated the points of different samples are, the closer the metabolite levels are. Conversely, the greater the degree of dispersion, the larger the difference in metabolite levels. Unsupervised principal component analysis (PCA) was employed to assess the overall disparity between the LJT and FLJT samples. PCA can clearly separated LJT and FLJT samples from QC samples (Figure 8), and the data points of QC group were highly concentrated, indicating that the collection process was highly reproducible. In addition, according to the first principal component (PC1) and the second principal component (PC2), the two groups of honeysuckle liquid samples were divided into different regions to highlight the influence of fermentation. PC1 (49.99%) and PC2 (14.89%) explained 64.88% of the total variance. The score chart showed that all samples were in the ellipse range, which showed that the samples had good repeatability, high data quality and no outliers. More importantly, LJT and FLJT distributed far away and showed obvious separation phenomenon, which indicated that fermentation could greatly change the types and contents of metabolites in honeysuckle liquid.

### 3.9 OPLS-DA metabolite of honeysuckle liquid before and after fermentation

**Figure 9:**
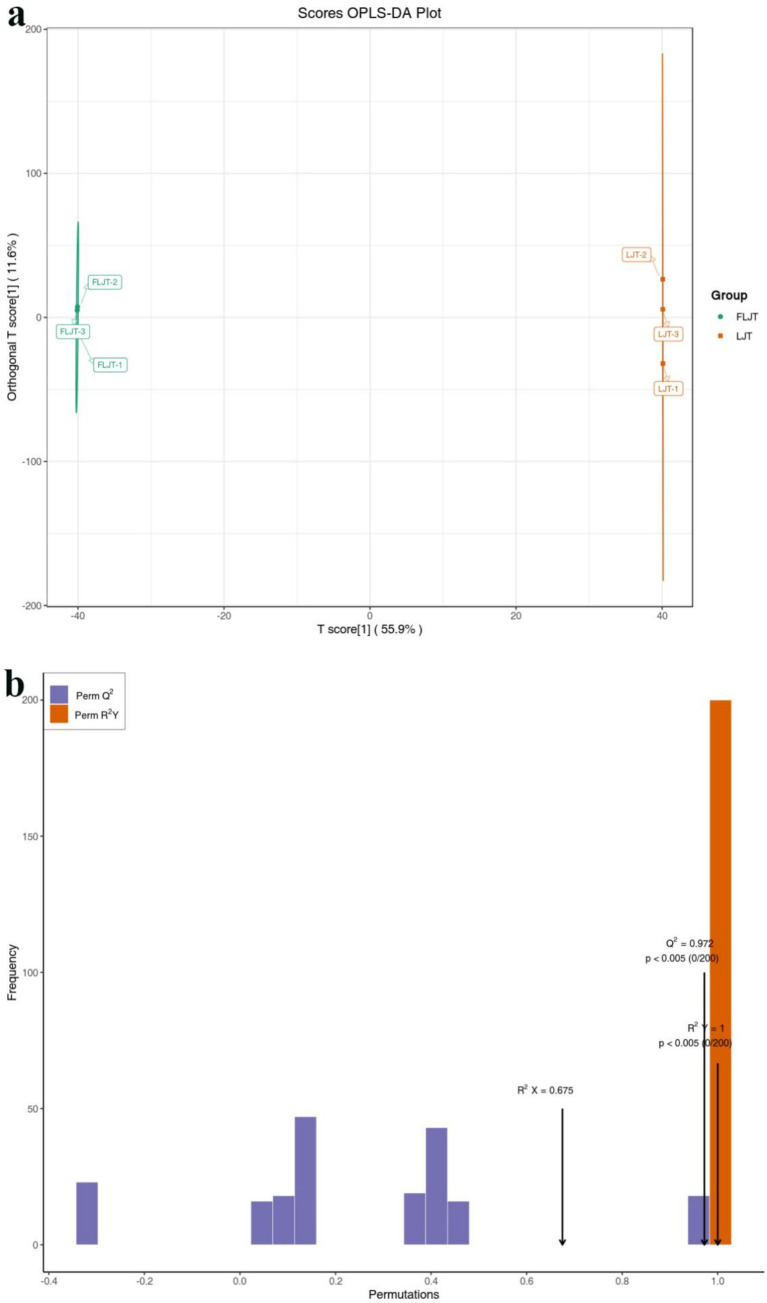
OPLS-DA score plot **(a)** and permutation test plot **(b)** of metabolites of LJT and FLJT.

The metabolic characteristics of LJT and FLJT were compared using the OPLS-DA model, the score diagram of which was shown in Figure 9a. From the score chart, it can be seen that FLJT was distributed on the negative axis of the abscissa axis, while LJT was distributed on the positive axis of the abscissa axis. The transverse distance between FLJT and LJT was far, and the separation phenomenon was obvious, which further verified the PCA analysis results and showed that there were obvious differences in the accumulation of metabolites between FLJT and LJT. In addition, it can be seen from the permutation verification chart that the scores of R2Y and Q2 were both greater than 0.9, and the *p* < 0.005 (Figure 9b).

**Figure 10:**
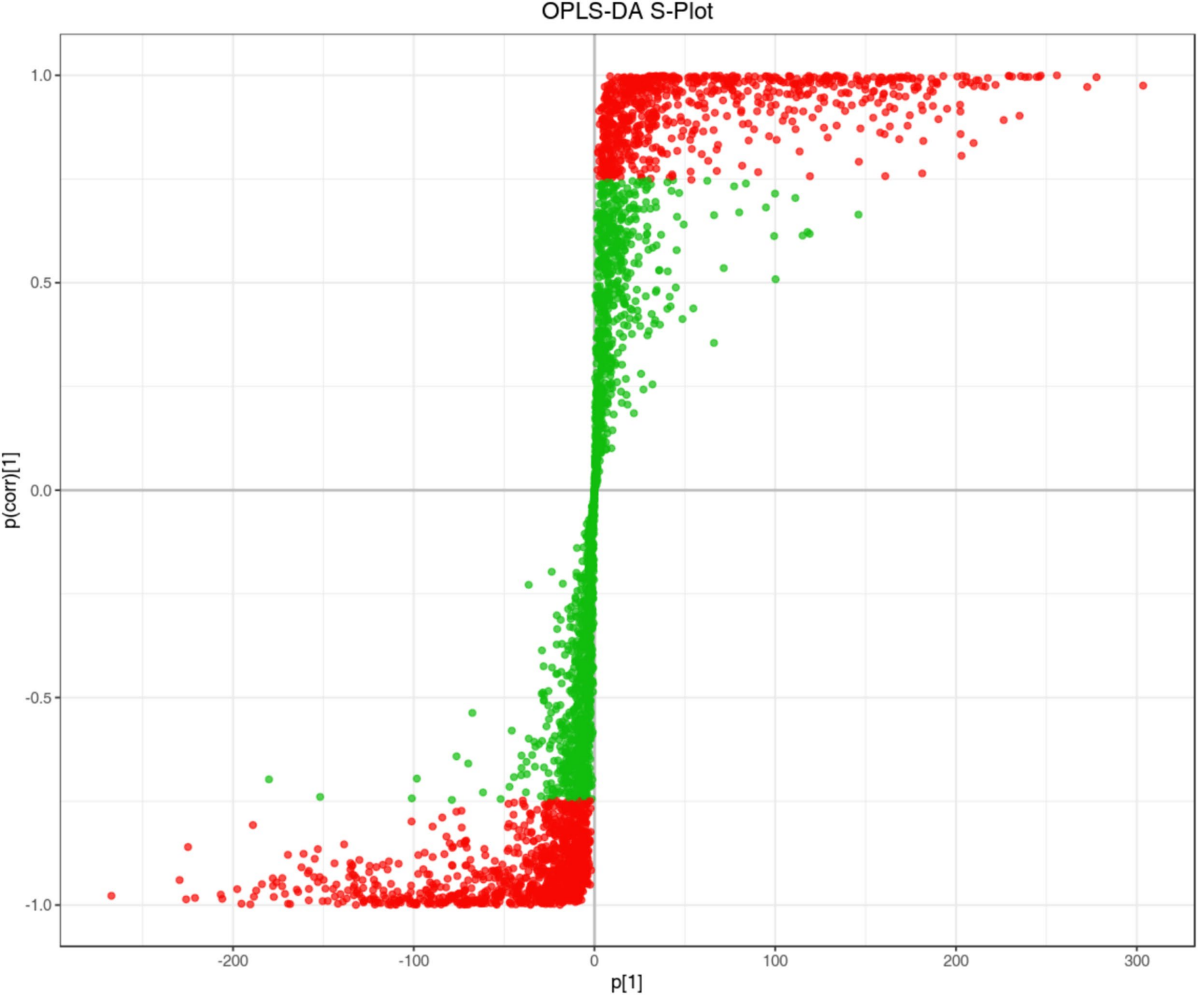
S-plot of OPLS-DA of LJT and FLJT metabolites. The red dot indicates the metabolites with VIP value greater than 1, the green dots represent metabolites with VIP values less than or equal to 1.

By using the VIP (Variable Importance in Project) value of OPLS-DA model, the differential metabolites among different species or tissues can be preliminarily identified, and combined with the univariate analysis of *p*-value or Fold Change (FC) value, the differential metabolites can be deeply screened. The S-plot diagram of OPLS-DA was to facilitate the discovery of potential differential metabolites of FLJT and LJT (Figure 10). The abscissa axis represented the covariance between principal components and metabolites, and the abscissa axis represented the correlation coefficient between principal components and metabolites. The more significant the difference of metabolites, the closer it was to the lower left corner and upper right corner of diagonal.

### 3.10 Screening and analysis of differential metabolites of honeysuckle liquid before and after fermentation

**Figure 11:**
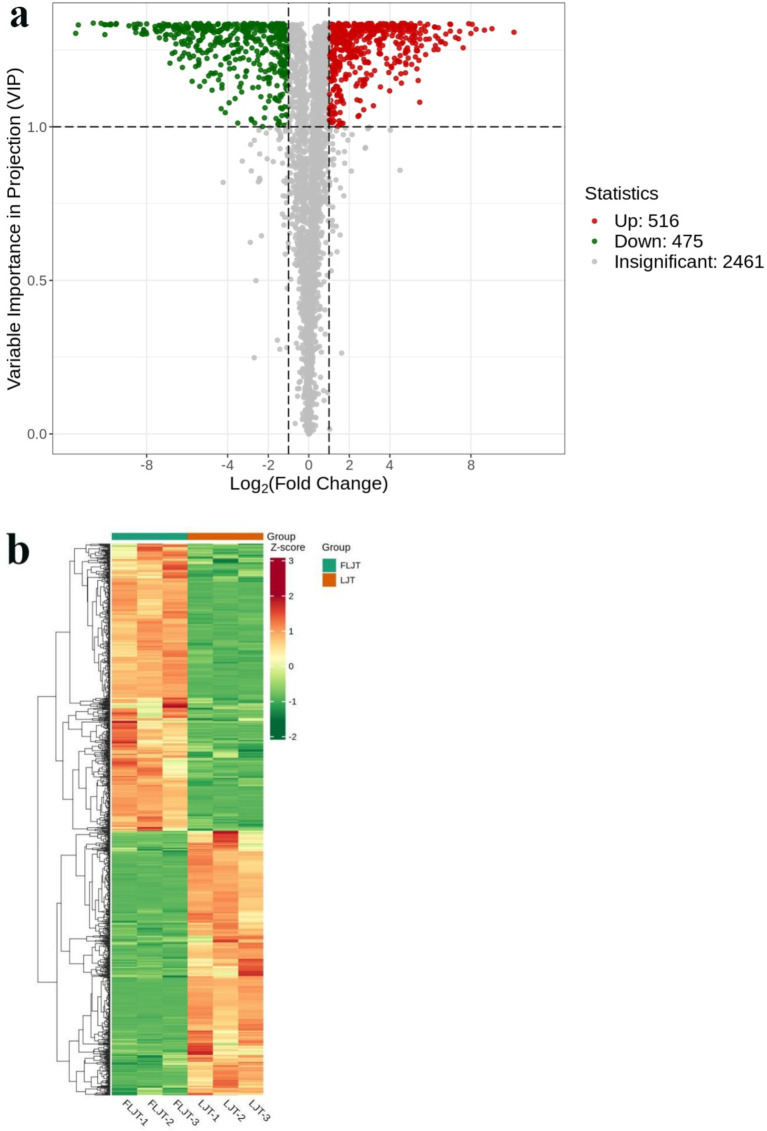
Volcanic map **(a)** and cluster heat map **(b)** of differential metabolites.

In this research, the combination of VIP (VIP > 1 and *p* < 0.05) and FC (FC > 2 and FC < 0.5) was used to screen the metabolites with significant difference, and the samples of honeysuckle liquid before and after fermentation were compared and analyzed. Volcanic map showed the overall distribution of differential metabolites in honeysuckle liquid (Figure 11a). Gray dots indicated that there was no significant difference in metabolites between FLJT group and LJT group, red dots indicated that metabolites in FLJT group were significantly up-regulated, and green dots indicated that metabolites in FLJT group were significantly down-regulated. The color of the cluster thermogram represented the relative content of metabolites in the sample (Figure 11b), where the color changed from green to red, indicating that the content of metabolites in the sample was up-regulated. The 991 kinds of differential metabolites were detected in honeysuckle liquid after fermentation, among which 475 kinds of up-regulated metabolites and 516 kinds of down-regulated metabolites. Through cluster analysis, it can be seen that fermentation can significantly change the content of differential metabolites in honeysuckle liquid, which showed that *L. acidophilus* in FLJT samples can promote or inhibit the production of various differential metabolites in the fermentation process, which provided a theoretical basis for the subsequent development of honeysuckle liquid fermentation products.

**Figure 12:**
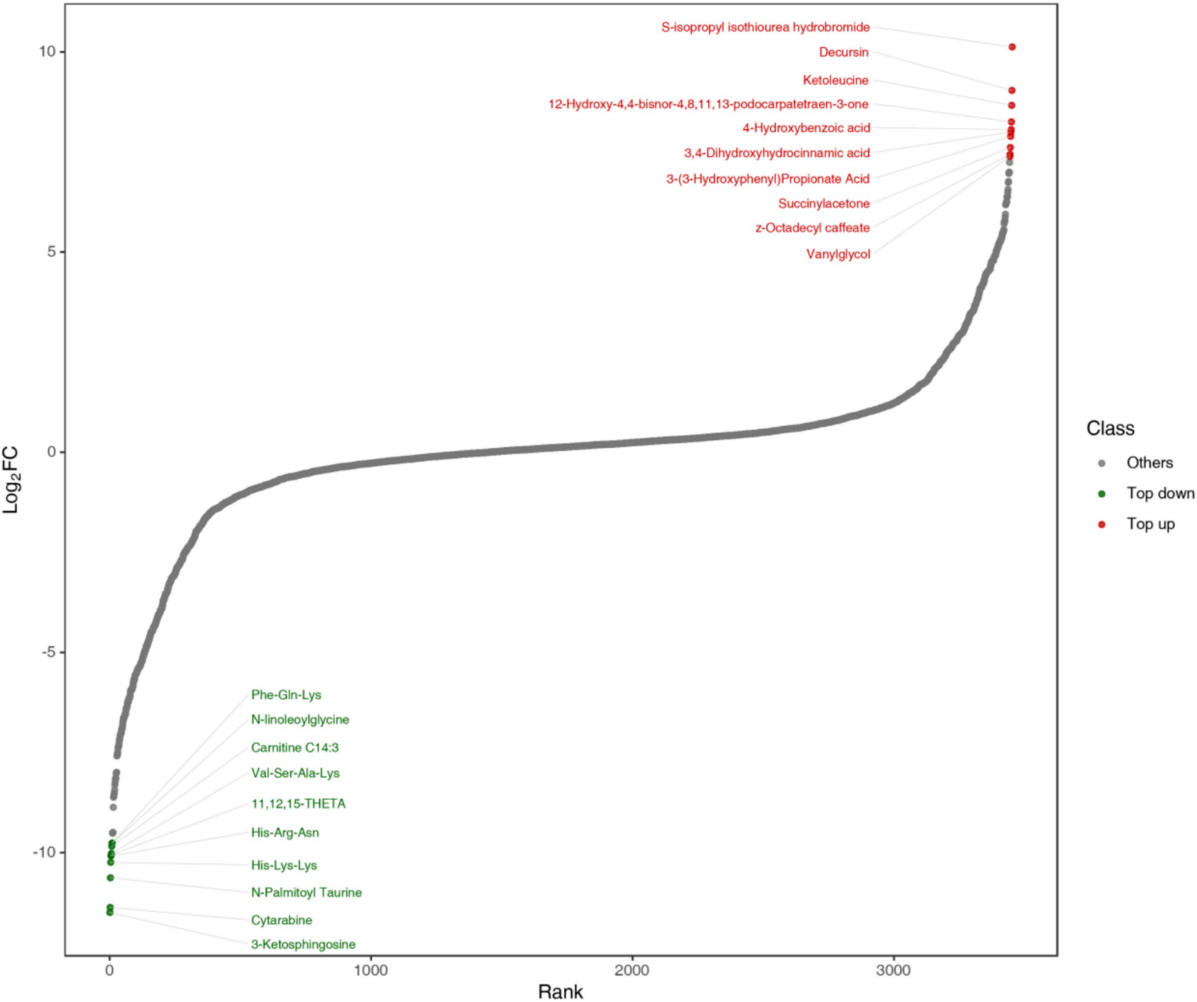
Distribution map of metabolites of LJT extract before and after fermentation.

To observe the overall metabolic differences more clearly and intuitively, the fold change (FC) of the metabolites in the two groups was calculated. Then, the metabolites were arranged in ascending order according to the FC value, and a distribution map of the metabolite content differences was plotted. The top 10 metabolites up and down were marked, each point represented a substance, the red point represented the top 10 substances up and the green point represents the top 10 substances down (Figure 12). As can be seen from Figure 12, S-isopropyl isothiourea hydrobromide in alcohols and amines, Decursin in lignans and coumarins, Ketoleucine in organic acids, 12-hydroxy-4, 4-bisnor-4, 8, 11, 13-podocarpatetraen-3-one in benzene and its derivatives, 4-hydroxybenzoic acid (4-hydroxybenzoic acid) and 3, 4-dihydroxyhydrocinnamic acid (dihydrocaffeic acid) in phenolic acids, and 3-hydroxyhydrocinnamic acid in organic acids (Octadecyl 4-dihydroxyphenyl) prop-2-enoic acid and Vanylglycols (3-methoxy-4-hydroxyphenylethylene glycol) in benzene and its derivatives were the metabolites with higher differential multiples in the up-regulated metabolites of fermented honeysuckle liquid; 3-Ketosphingosine in alcohols and amines, Cytarabine (cytarabine) in nucleotides and their derivatives, N-Palmitoyl Taurine (N-palmitoyl taurine) in organic acids, His-Lys-Lys (histidine-lysine-lysine) and His-Arg-Asn (histidine-arginine-asparagine) in amino acids and their derivatives, 11, 12, 15-THETA in lipids, Val-Ser-Ala-Lys in amino acids and their derivatives, Carnitine C14:3 (carnitine C14:3) in fatty acyl, N-linoleoylglycine and Phe-Gln-Lys in Amino acids and their derivatives were a metabolite with a higher differential ratio among down-regulated metabolites.

### 3.11 Enrichment analysis of KEGG metabolic pathway

**Figure 13:**
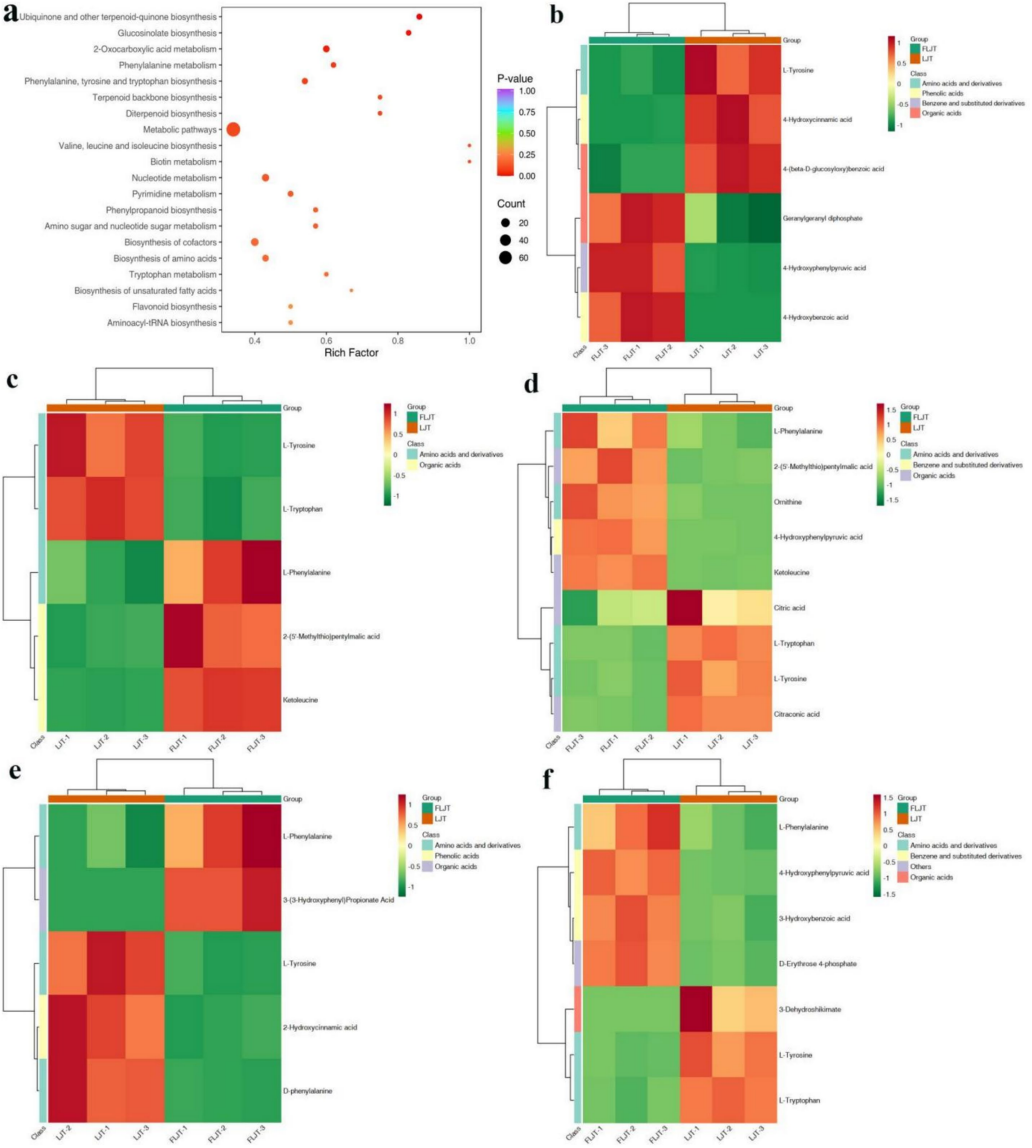
KEGG enrichment map and heat map of differential metabolites. KEGG enrichment map **(a)**. Ubiquinone and other terpenoid-quinone biosynthesis **(b)**. Glucosinolate biosynthesis **(c)**. 2-oxocarboxylic acid metabolism **(d)**. Phenylalanine metabolism **(e)**. Phenylalanine, tyrosine and tryptophan biosynthesis **(f)**.

The results showed that 991 differential metabolites were enriched into 79 metabolic pathways, and the first 20 major metabolic pathways were shown in Figure 13a. Among them, the top five metabolic pathways are: biosynthesis of ubiquinone and other terpenoid quinones (Figure 13b), in which six differential metabolites such as isoprene pyrophosphate, 4-hydroxyphenylpyruvate and 4-hydroxycinnamic acid were found; Biosynthesis of glucosinolates (Figure 13c), in which five differential metabolites such as L-phenylalanine, 2-(5-methylthio) amyl malic acid and 4-methyl-2-oxo valeric acid were found; 2-oxocarboxylic acid metabolism (Figure 13d), in which 9 differential metabolites such as L-phenylalanine, 2-(5-methylthio) amyl malic acid, ornithine, 4-hydroxyphenylpyruvate and 4-methyl-2-oxo valeric acid were found; Phenylalanine metabolism (Figure 13e), in which five differential metabolites such as L-phenylalanine and 3-(3-hydroxyphenyl) propionic acid were found; Biosynthesis of phenylalanine, tyrosine and tryptophan (Figure 13f). Seven differential metabolites such as L-phenylalanine, 4-hydroxyphenylpyruvate, m-hydroxybenzoic acid and D-erythritose-4-phosphate were found in this pathway.

## 4 Discussion

Lactobacillus is an important part of probiotics, which has probiotic functions such as regulating intestinal flora balance, inhibiting cholesterol absorption, lowering blood lipid and blood sugar, and is often positively correlated with the number of viable bacteria (Ran et al., 2024b). However, different LAB have different stress tolerance during fermentation, and pH, osmotic pressure and composition of fermentation substrate will affect the growth of LAB. The higher viability of LP-1 and LA may be due to their stronger ability to utilize carbon sources in honeysuckle liquid, which is consistent with the research results of Liu et al. (2022). The colony count of all strains remained above 8 log CFU/mL, which met the minimum viability level that probiotics might have beneficial effects on intestinal health after passing through digestive tract.

LAB fermentation usually leads to the decrease of pH of food substrate due to acid production (Tkacz et al., 2020). The pH of fermented honeysuckle liquid decreased significantly from 5.41 to 3.29–4.96, which was caused by the accumulation of organic acids produced by LAB fermentation. LAB utilized the carbon source of honeysuckle fermentation broth and produce organic acids such as lactic acid and pyruvate, which will reduce the pH of honeysuckle broth. The pH of honeysuckle liquid fermented by LP-1 and LA was the lowest, which could be attributed to the high content of organic acids produced by LP-1 and LA. Relevant studies have shown that the ability of different LAB to produce acid by using carbon source is different in the fermentation process (Sun et al., 2022; Duan et al., 2023).

The effect of LAB fermentation on color characteristics can be expressed by L* value, a* value, b* value and *Δ* E value. The color change of honeysuckle liquid may be caused by the hydrolysis and destruction of plant cell wall during fermentation, which reduces the size of plant tissue fragments and increases the release of phytochemicals (Lu et al., 2020). LAB fermentation makes the color of honeysuckle liquid more bright, especially the L* and B* values of honeysuckle liquid fermented by LA are the highest, which shows that the brightness and yellowness of honeysuckle liquid fermented by LA are the strongest. At the same time, the difference of Δ E between LA fermented honeysuckle liquid and control was the largest, and Δ E was in a more significant range (ΔE > 6.0).

Polyphenols are closely combined with cellulose matrix in food, which will affect its bioavailability and biological efficacy. Therefore, converting these polyphenol polymers into simpler compounds by enzymatic hydrolysis or fermentation is a strategy to improve the bioavailability of phenolic compounds (de Souza et al., 2019). LAB fermentation can not only release more soluble conjugated phenolic compounds from the cell wall of plant substrate, but also transform complex phenolic substances into simpler compounds and metabolites with low molecular weight, high bioavailability and biological activity through enzymatic hydrolysis of glycosyl hydrolase, phenolic acid decarboxylase, reductase and esterase (Mantzourani et al., 2019; Shang et al., 2022). The difference of phytochemical content of different kinds strains of LAB after fermentation may be due to their individual adaptability and their ability to produce hydrolase. Some studies have pointed out that LA fermentation can increase the content of polyphenols in fermentation broth and change the composition of polyphenols (Chen et al., 2022). In this study, all 9 strains of LAB can significantly increase the total polyphenols content of the honeysuckle liquid, and hydrolyze complex phenolic compounds into small molecular phenols. At the same time, organic acids produced in the fermentation process can prevent the degradation of phenolic substances (Filannino et al., 2018). It was found that the bioconversion rate of polyphenols increased during fermentation because the specific metabolism of LAB took place polyphenol deglycosylation reaction, which converted macromolecular polyphenols into small molecular polyphenols. This study found that the fermentation of most LAB increased the total flavonoid content of honeysuckle liquid. The increase of flavonoid content may be due to the decomposition of complex high molecular weight phenolic compounds into simpler flavonoids by hydrolases during fermentation, and specific glycosyl hydrolases can convert flavonoid glycosides into corresponding glycoside ligands (Filannino et al., 2018). The increase of chlorogenic acid content in honeysuckle liquid after LAB strain fermentation can be explained by chemical decomposition of anthocyanins (Ryu et al., 2019). Cheng et al. (2016) found that the main metabolites of mulberry anthocyanins degraded by LAB are phenolic compounds and chlorogenic acid.

According to related reports, the transformation of phenolic compounds and other substances will affect its biological activity, which indicates that LAB fermentation may change the functional characteristics of honeysuckle liquid (Qi et al., 2021). Chlorogenic acid has antioxidant activity, and its molecules contain carboxyl and hydroxyl groups, which can form hydrogen radicals and have the ability to scavenge free radicals (Tošović and Marković, 2018). Different antioxidant activity mechanisms lead to different increasing trends of antioxidant capacity of the four methods. DPPH radical scavenging of abnormal electrons based on nitrogen atoms is reduced by accepting hydrogen atoms in antioxidants, hydroxyl radical scavenging is based on giving hydrogen atoms to hydroxyl radicals to stabilize their activity, superoxide anion radical scavenging is based on trapping superoxide anion radicals and reacting with them to neutralize their activity, and reducing ability is to evaluate the ability to provide electrons to reduce Fe^3+^ to Fe^2+^ to terminate radical chain reaction (Kwaw et al., 2018). The increase of antioxidant capacity of honeysuckle liquid is consistent with the increase trend of polyphenols, total flavonoids and chlorogenic acid, which may be the result of the synergistic action of these bioactive substances. The increase of antioxidant capacity may be related to the increase of phenolic compounds and flavonoids produced by microbial hydrolysis during fermentation. In addition, during the fermentation process, LAB produce organic acids some extracellular enzymes, such as cellulase, galacturonase, xylanase, etc. These components destroy the structure of cell wall, resulting in the release and dissociation of various antioxidant compounds.

A-glucosidase hydrolyzes starch and other polysaccharides to produce glucose, which can control short-term hyperglycemia by inhibiting its activity, and is an effective way to regulate blood sugar (Ali et al., 2024). The increase of *α*-glucosidase inhibition ability is related to the content of phenols and lactic acid, phenolic compounds, as inhibitors of α-glucosidase, can inhibit the decomposition of carbohydrates, thus stabilizing the blood sugar level *in vivo* (Ankolekar et al., 2012). The honeysuckle fermentation broth fermented by LAB has significantly enhanced inhibitory ability against α-glucosidase. Sun et al. (2022) fermented pumpkin juice by LAB strain, and found that the inhibition rate of α-glucosidase in fermented pumpkin juice increased from 26.97 to 36.39–48.35%. Zhang et al. (2021) reported that LP fermentation significantly increased the inhibition rate of α-glucosidase in blueberry juice, reaching about 45.00% after fermentation, which was consistent with the results. Therefore, the improvement of α-glucosidase inhibition ability may be caused by the increase of chlorogenic acid, total phenols and flavonoids in honeysuckle fermentation broth, and the hypoglycemic ability of honeysuckle fermentation broth fermented by different strains varies with the content of these bioactive substances.

Pearson correlation analysis was carried out between active substances (total phenols, total flavonoids and chlorogenic acid) and functional characteristics (antioxidant activity and α-glucosidase inhibition) of honeysuckle liquid after fermentation. The results indicated that the increase of DPPH radical, hydroxyl radical and superoxide anion radical scavenging activity, reducing ability and α-glucosidase inhibition ability after honeysuckle fermentation was due to the increase of total phenol, total flavonoids and chlorogenic acid content. Quality control samples are prepared by mixing sample extracts, so as to analyze the repeatability of samples under the same treatment method. QC samples were analyzed according to the above conditions of chromatography and mass spectrometry. The consistency of metabolite extraction and detection can be evaluated by comparing the mass spectrometry detection of QC samples with different quality control by overlapping display analysis total ion flow diagram (TIC diagram). From the correlation analysis results of positive ion and negative ion mode QC samples, it can be seen that QC samples have high correlation and high data quality. Although PCA can effectively extract the main information of metabolites, it is not sensitive to variables with less correlation. OPLS-DA is different from PCA in that it can use partial least squares regression to establish the relationship model between metabolite expression and sample category, which is beneficial to find key symbolic differential metabolites. Therefore, using OPLS-DA model to analyze metabolomics data can further analyze the differences among components. In OPLS-DA score chart, the abscissa axis T reveals the differences between groups of samples, and the ordinate axis T reveals the differences within sample groups. Each point represents a sample, and the experimental groups are distinguished by different scatter shapes and colors. The further the horizontal distance between samples, the greater the difference between sample groups; The closer the longitudinal distance is, the better the repeatability within the group. In addition, parameters R2X, R2Y and Q2 are needed to quantitatively evaluate the stability and reliability of OPLS-DA model. Among them, R2X and R2Y represent the interpretation rate of X matrix and Y matrix, and Q2 represents the prediction ability. The closer the values of R2X, R2Y and Q2 are to 1, the more stable and reliable the model is; When Q2 value is greater than 0.5, it is considered as a valid model, and when Q2 value is greater than 0.9, it is considered as an excellent model. Permutation verification evaluates the model performance with 200 random data permutations. In the permutation verification diagram, the horizontal axis represents R2Y and Q2 values, and the vertical axis represents the frequency of classification effect (Yue et al., 2019). Usually, when *p <* 0.05, the model is the most ideal. The results indicated that the model is accurate and reliable, and can be used for subsequent data analysis.

Related studies have proved that 4-hydroxybenzoic acid and 3, 4-dihydroxyhydrocinnamic acid have certain antioxidant capacity and can reduce oxidative stress response. Dos Santos et al. (2021) found that 3, 4-Dihydroxyhydrocinnamic acid is also a coenzyme A ester, which participates in the formation of rosmarinic acid. In addition, 4-hydroxybenzoic acid and 3, 4-dihydroxyhydrocinnamic acid are considered as active ingredients with various uses because of their antioxidant properties, and are often used in the formulation of dietary supplements and food gels. Decursin has anti-inflammatory and analgesic effects, and recent studies have found that it also has antioxidant properties (Ko et al., 2020). Therefore, the increase of 4-hydroxybenzoic acid, 3, 4-dihydroxyhydrocinnamic acid and decursin in the fermented honeysuckle liquid plays an important role in improving the antioxidant capacity of honeysuckle liquid. In addition, amino acids and other substances showed significant down-regulation in the distribution map of metabolite content difference. Although amino acids themselves do not belong to volatile flavor components in fermented foods, they can be further converted into alcohols, aldehydes, esters and sulfur compounds, which can greatly enhance the taste and flavor of fermented foods. Amino acids play a vital role in the fermentation process. Microorganisms can accelerate the fermentation cycle by using amino acids as nitrogen sources for maintaining growth and reproduction. These may be the reason why the content of amino acids and other substances in honeysuckle liquid decreased during fermentation.

Microbial fermentation is a very complex metabolic process, which is regulated by many substances, factors and biochemical reactions, and can not be judged only by the content of a certain substance, so it is necessary to further analyze its metabolic pathway. According to the screening criteria, the pathway enrichment analysis of differential metabolites was carried out through KEGG database. The function of this substance can be inferred according to the differential metabolites, and the differentially up-regulated substances in fermented honeysuckle liquid may have positive regulation effect. L-phenylalanine is significantly increased in the first five metabolic pathways, it’s a kind of aromatic amino acid with pharmacological activity and one of the essential amino acids for human body. It is mainly used in sweeteners, flavoring agents and health products in food industry. L-phenylalanine is mainly synthesized through the transamination of pyruvic acid. In this process, glutamate pyruvate transaminase (GPT) catalyzes the amino transfer reaction between pyruvic acid and glutamic acid, generating L-phenylalanine and *α*-ketoglutaric acid. When the level of glutamic acid is high within the cell and there is an abundant energy supply, this reaction will proceed in the direction of generating L-phenylalanine (Ma et al., 2019; Wu et al., 2018). Organic acids and phenolic compounds are important bioactive substances in honeysuckle. Phenolic compounds mainly include flavonoids, phenolic acids and terpenoids. In the first five metabolic pathways, 4-hydroxycinnamic acid, a phenolic compound, has an aromatic ring structure and can be used as a precursor in the biosynthesis of anthocyanins and other flavonoids, which may also be one of the reasons for the increase of total flavonoids in honeysuckle liquid caused by fermentation. In addition, 4-hydroxycinnamic acid has the effects of protecting nerves and resisting cancer (Wang et al., 2018; Winter et al., 2017). It is worth noting that 4-hydroxycinnamic acid can be used as a food additive to prevent food deterioration. Muangsri et al. (2022). The results of enhanced antioxidant activity of 4-hydroxycinnamic acid loaded on sodium alginate and polyvinyl alcohol hydrogel composites verify this view. In addition, flavonoid biosynthesis was also included in the first 20 significantly changed metabolic pathways, and six differential metabolites such as chlorogenic acid, catechin and quercetin were found in this pathway. Among them, chlorogenic acid, as the main functional active substance in honeysuckle fermentation broth, was enriched in flavonoid biosynthesis of differential metabolic pathway, which also increased significantly. The chlorogenic acid also plays a beneficial role in the prevention and treatment of cardiovascular diseases, malignant tumors and diabetes (Clifford et al., 2020). Therefore, the improvement of antioxidant activity and α-glucosidase inhibition of fermented honeysuckle liquid may be related to the increase of phenols and flavonoids including chlorogenic acid.

## 5 Conclusion

The 10 strains of different LAB were used to study the viable bacteria, pH, color characteristics, total phenol content, flavonoids content, chlorogenic acid content, antioxidant activity and α-glucosidase activity of honeysuckle liquid. *L. acidophilus* zrx02 was the most effective strain among the 10 kinds strains of LAB used for fermenting honeysuckle liquid. The 991 metabolites with significant differences were screened by metabolomics analysis, which were enriched into 79 metabolic pathways. Fermentation by *L. acidophilus* zrx02 could promote the synthesis and accumulation of secondary metabolites in honeysuckle liquid. Fermentation increased the content of small-molecule phenolic substances and improved the digestion and absorption characteristics of honeysuckle polyphenols, making it possible for honeysuckle polyphenols to exert their functional properties in the body. The research analyzed the mechanism of polyphenol biotransformation from the perspective of metabolomics and provided new ideas for the intensive processing of honeysuckle.

## Data Availability

The raw data supporting the conclusions of this article will be made available by the authors, without undue reservation.
